# Enhancing the Use of Vehicle Alcohol Interlocks With Emerging Technology

**DOI:** 10.35946/arcr.v36.1.08

**Published:** 2014

**Authors:** Robert B. Voas

**Affiliations:** Robert B. Voas, Ph.D., is a senior research scientist at the Pacific Institute for Research and Evaluation, Calverton, Maryland.

**Keywords:** Alcohol use, abuse, and dependence, drinking and driving, driving under the influence (DUI), impaired driver, drinking and driving laws, traffic safety, blood alcohol content (BAC) tests, electronic monitoring of offenders, impaired-driving law enforcement, vehicle alcohol interlock device, interlock programs, diagnostic vehicle device, monitoring device, electronic health technology

## Abstract

Among the earliest applications of health technologies to a safety program was the development of blood alcohol content (BAC) tests for use in impaired-driving enforcement. This led to the development of miniature, highly accurate devices that officers could carry in their pockets. A natural extension of this technology was the vehicle alcohol interlock, which is used to reduce recidivism among drivers convicted of driving under the influence (DUI) by requiring them to install the devices (which will not allow someone with a positive BAC to drive) on their vehicles. While on the vehicle, interlocks have been shown to reduce recidivism by two-thirds. Use of these devices has been growing at the rate of 10 to 15 percent a year, and there currently are more than 300,000 units in use. This expansion in the application of interlocks has benefited from the integration of other emerging technologies into interlock systems. Such technologies include data systems that record both driver actions and vehicle responses, miniature cameras and face recognition to identify the user, Wi-Fi systems to provide rapid reporting on offender performance and any attempt to circumvent the device, GPS tracking of the vehicle, and more rapid means for monitoring the integrity of the interlock system. This article describes how these health technologies are being applied in interlock programs and the outlook for new technologies and new court sanctioning programs that may influence the growth in the use of interlocks in the future.

## Blood Alcohol Content (BAC) Technology in Impaired-Driving Enforcement

One pathway for the development of new electronic health technologies originates from two unexpected sources: the literature on the enforcement of drinking-and-driving laws and the development of vehicle alcohol interlock devices. Traffic safety was one of the earliest areas to apply electronic technologies in health and prevention programs. The first tests for assessing alcohol in the human body were collected in the late in 19th century (American Medical Association [AMA] 1970). In the first half of the 20th century, Widmark (1932) described the body’s system for the absorption and elimination of alcohol. Heise (1934) in the United States and Goldberg (1943) in Sweden developed the evidence relating BAC to driving-related skills that provided the theoretical basis for the use of alcohol tests for forensic purposes. This, in turn, led to the rapid development of accurate methods for assessing alcohol in blood and urine. Reviews of this technology are available from several sources (Casier and Delaunois 1947; Harger and Hulpier 1936) and most recently from Jones (2000). The first use of alcohol tests by the courts is unknown; however, there is a record of appellate court review in 1937 (AMA 1970). The first State law establishing the use of forensic tests for alcohol was enacted by Indiana in 1939. The first State to establish a specific BAC level (0.15 percent) as a per se violation was Nebraska. Currently, all 50 States have per se laws providing that operating a vehicle with a 0.08 BAC is illegal. By mid-century, laboratory methods for measuring alcohol in blood, urine, and breath had been developed (Smith 1965). The Grand Rapids study (Borkenstein et al. 1974) demonstrated the strong relationship between BAC and crash involvement and further strengthened the use of chemical tests in the prosecution of impaired-driving offenders.

Forensic tests for alcohol are important for the enforcement of drunk-driving laws, because motorists significantly impaired by alcohol do not necessarily manifest the signs of intoxication such as slurred speech and loss of balance that are recognized by juries. Depending entirely on the police officer’s description of the driver’s behavior seriously limits the strength with which impaired-driving laws can be enforced. The development of chemical tests for intoxication played a strong role in increasing the effectiveness of the criminal justice system in prosecuting impaired drivers. However, wet chemistry analysis involved inconvenient collection systems (e.g., phlebotomists for blood sample collection) and required shipping samples to State laboratories for analysis with significant costs and, in some cases, sub-stantial delays in receiving the results. Two technological developments in the 1950s substantially reduced this problem with the use of BAC tests in impaired-driving enforcement: the Borkenstein Breathalyzer and the fuel-cell sensor. The Breathalyzer (Borkenstein and Smith 1961) was a simplified photometric analysis device (about the size of a typewriter) that could be operated by a police officer with modest training. Developed in 1961, it provided, for the first time, a method for collecting forensic-quality BAC data in the police station at low cost and with immediately available results. This greatly enhanced the speed with which driving-under-the-influence (DUI) offenders could be processed and the quality of evidence that could be brought to court.

With the increased role of BAC tests in the prosecution of impaired drivers, officers in the field needed a reliable method of estimating BAC. In the early 1970s, researchers developed hand-held “preliminary breath testers” (PBTs) based on fuel-cell sensor technology (an offshoot from the space program), which detects alcohol with excellent accuracy and precision. The fuel cells’ small size made it possible to incorporate them into use with PBTs the size of a cigarette package, which officers could carry in their pocket and use to screen drivers for whom there was evidence of drinking. The small size of fuel cells also permitted the development of passive alcohol-sensing systems that could be incorporated into an officer’s flashlight (Ferguson et al. 1995). Heavy drinking could be detected by holding the flashlight within 4 to 6 inches of the driver’s face and sampling the ambient air.

## Development of the Vehicle Alcohol Ignition Interlock

The technological developments described above, which resulted in State laws specifying BAC limits and in the development of technology to enforce those limits, also led to the development of the vehicle alcohol interlock safety system to prevent a driver with a measurable BAC from starting his or her car (Compton 1988; Mayer 2014). Vehicle interlocks incorporate fuel-cell breath-testing systems to ensure the driver has a zero BAC (generally defined as BAC < 0.02). The driver must use the interlock system each time he or she starts the car and at random intervals during driving. The first vehicle demonstrating such a system was shown to the Department of Transportation in 1970 (Voas 1970). However, unlike the PBT, which was used by police who could ensure its maintenance and integrity, the vehicle interlock was intended for managing impaired-driving offenders who might be expected to attempt to circumvent the device. It required several years to develop protective systems that would prevent or, at a minimum, reveal efforts to tamper with or circumvent the interlock. As a result, the widespread use of vehicle interlocks did not begin until the 1990s, and the first national model standard for interlock devices was not issued until 1992 (Federal Register 1992; National Highway Traffic Safety Administration 2013). Since that time, however, their use has increased by 10 percent to 15 percent per year, with over 300,000 currently in use with DUI offenders (Roth 2012).

Although the fuel-cell sensor is an established technology, vehicle interlock systems continue to grow more effective and efficient through the integration of other emerging technologies. Early models obtained data on offender performance only at monthly service contacts. Now, with the growth of the Internet, this technology has fostered a trend for interlock companies to provide daily or even real-time data transmission to monitoring agencies. Advances in photography and facial imaging enable better identification of the driver providing the breath sample. GPS allows monitoring of the offender’s driving and location when a breath test is being provided. These enhancements are broadening the application of vehicle interlocks to court-based recovery programs such as those implemented by special DUI courts (Voas et al. 2011).

## Evidence for the Effectiveness of Interlocks

The basic research findings regarding the interlock are well understood (Marques and Voas 2010):

- Generally, only 10 percent to 20 percent of offenders will choose the interlock option over short-term license suspension (Marques and Voas 2010; Voas et al. 1999, 2001). The 2013 estimated installation rate is 20 percent (Roth 2012; United States Government Accountability Office 2014). However, a higher installation rate can be produced if the alternative to installation, such as electronically monitored house arrest, is less desirable than the interlock (Roth et al. 2009; Voas et al. 2001).
- Strong evidence exists for their effectiveness while on the vehicle. Two meta-analyses of interlock evaluations have demonstrated highly similar results indicating that while on the vehicle, interlocks reduce recidivism by approximately 64 percent (Elder et al. 2011; Willis et al. 2004). With one exception (Rauch et al. 2011), all evaluations have agreed that this benefit does not carry over into the period beyond the removal of the interlock (Elder et al. 2011; Willis et al. 2004).
- Substantial evidence shows that the frequency of an offender being prevented from starting the vehicle because of lockouts predicts recidivism following the removal of the unit from the offender’s vehicle (Marques et al. 2001, 2003*a*,*b*).

Based on this evidence, the major efforts to extend the effectiveness of interlocks have focused on the following:

- Increasing the number of offenders who install the units;
- Closer monitoring of performance while on the interlock to increase the current 64 percent recidivism benefit during that period (Voas et al. 2013; Zador et al. 2011);
- Extending the time on the interlock for offenders who perform poorly (have an excessive number of lockouts) (Mayer 2014); and
- Implementing health promotion programs tied to the interlock while the units are in place on the vehicle (Timken and Marques 2003*b*; Timken et al. 1995), which might extend the benefit following removal (Marques and Voas 2013).

## Applying New Technology to Improve Interlock Effectiveness

This article describes how new electronic health technologies are being applied or can be applied to enhance the use of vehicle interlocks and achieve the goals listed above. The core technological elements of the interlock are the miniaturized fuel-cell sensor, the technology for inserting the device into the vehicle ignition sequence, the digital program that manages rolling retests, and other functions of the device and the circumvention prevention system. This article covers technological developments relevant to health and safety that are influencing the application of the interlock to the management of DUI offenders. These fall into two categories: (1) items that are integrated into the interlock units, which include the interlock data logger system, photo or facial identification of the user, and Wi-Fi data transfer systems, and (2) new technological monitoring programs that are producing alternatives to the use of the interlock such as BAC sensors attached to or carried by individual offenders to monitor drinking on a 24/7 basis and GPS systems or similar sensing technology currently under development to monitor driving. Alternative programs, although they may compete with the interlock and limit its use, are also relevant to the spread of interlocks because they are being used to motivate offenders to install interlocks to avoid enforced abstinence.

## Use of Electronic Health Technologies to Monitor and Manage DUI Offenders’ Behaviors

When the first interlock-equipped vehicle was displayed at the Department of Transportation in 1970, it seemed to offer a solution to the prevalent concern that license suspension, while protecting the public against the risk DUI offenders represented to other drivers, would adversely affect the employment of offenders and thus their family’s welfare. Although DUI offenders might be expected to welcome the opportunity to install an interlock, given that the alternative is license revocation or suspension, surprisingly, most studies have noted low participation rates when offenders are offered the opportunity to install a unit. Elder and colleagues (2011), in their meta-analysis of 17 studies, noted a wide variation in offender participation from less than 1 percent to 64 percent, with a median participation rate of only 13 percent. Low rates generally were experienced when offenders had an option to install an interlock or remain suspended. Two studies (Roth et al. 2009; Voas et al. 2001) demonstrated that making the alternative to installation more punitive by requiring home confinement or jail as the alternative to the interlock increased installation rates substantially. Thus, one method for encouraging installation is to make the alternative less desirable than simply being unable to drive legally. This need to motivate offenders to install interlocks has led States to enact legislation mandating interlocks for DUI offenders. However, the power of the courts to enforce such laws has been limited by the requirement that the offender possess a car on which the device can be installed. Substantial numbers of DUI offenders have access to vehicles not registered in their own names for which the court cannot force installation. Absent a vehicle, the offender generally is left with a suspended license and an incentive to drive illicitly, and research indicates that up to 80 percent do drive while suspended (McCartt et al. 2002; Ross and Gonzales 1987). Given this resistance to interlocks, alternative methods for controlling the driving of those offenders who currently avoid the interlock are needed. The potential traditional penalty alternatives—fines and community service—do not prevent driving, and jail is too disruptive to the offender and expensive for the State to be used as a motivator for installing interlocks.

## Alternative Methods of Detecting Alcohol Use

Recently, the development of practical electronic technology for continuous remote-monitoring BAC is offering an alternative to the interlock that is applicable to all offenders. A major advance in a practical system for monitoring the drinking of DUI offenders in order to prevent impaired driving has resulted from the research on transdermal-sensing systems that detect the 1 percent of alcohol eliminated through the skin (Swift 2003). This technology has been engineered into systems that can be attached to the skin with an ankle or wrist bracelet that can be worn for several months by DUI offenders to monitor drinking. These units detect and record small increases in alcohol elimination through the skin that occur from 1.5 to 2 hours after consumption. The data are downloaded to a modem in the offender’s home and transmitted daily to the monitoring authority. Currently, the most widely used device is the Secure, Continuous, Remote Alcohol Monitoring (SCRAM™) produced by Alcohol Monitoring Systems, Inc. (Barnett et al. 2014; Marques and McKnight 2009; Sakai et al. 2006). As of the date of this article, the manufacturer reports that more than 50,000 SCRAM™ units currently are in use in 1,800 courts in 48 States. A competing remote electronic BAC-sensing system for monitoring alcohol use is the use of small portable breath-test devices with photo or facial recognition for verifying the person providing the breath sample. One example of this type of instrument is the IN-HOM™ system produced by Smart Start, Inc. It can be set to require multiple breath tests during a day to detect drinking. As with the transdermal devices, the data are recorded and transmitted electronically daily. These two types of systems offer the court an alternative to the interlock for controlling impaired driving through the prevention of drinking.

## Potential Alternative to Monitoring BAC: Activity Tracker that Detects Driving

As noted, a significant limitation in motivating DUI offenders to install interlocks is the weakness of license suspension as an alternative to be avoided. Enforcement of licensing laws is difficult because officers must have a reason to stop a driver to check the license. That unlicensed drivers perceive that the risk of apprehension is low is evidenced by the high rates of illicit driving (McCartt et al. 2002; Ross and Gonzales 1987). There is a need to develop a remote system of monitoring driving similar to that provided by the SCRAM™ device. The development of such a remote system for supervising driving that can be applied to all DUI offenders is on the horizon. Initial studies have shown that the typical movement of the feet when driving has a unique pattern that can be distinguished from simply riding in a vehicle or other activities such as walking and bicycle riding. A driving-monitoring system (NO-DRIV™) uses an ankle bracelet with miniature accelerometers on each leg and is currently being developed by Smart Start, Inc. The device is designed to detect and to record driving and transmit a report within 24 hours to the monitoring station. Proofof-concept testing has been completed, but feasibility testing remains to be conducted. Thus, it is likely that the NO-DRIV™ or a similar system will become available in the near future, providing the court with another alternative to the interlock: the prevention of driving.

## Potential Full-Service Systems Approach to Implementing Interlock Programs

With the development of these new electronic health technologies, there is an opportunity to take a systems approach to managing the driving of DUI offenders. In addition to the interlocks, which prevent the combination of drinking with driving, the courts will have the capability of preventing any drinking that results in significant driver impairment or preventing any driving. The availability of these three technologies will provide the court with flexibility in applying a sanction best suited to the offender’s characteristics. Employed as a system, remote monitoring of drinking or of driving become viable alternatives to the interlock for protecting the driving public. Moreover, given the likelihood that some, perhaps most, DUI offenders will prefer the interlock to either of those options, it is likely that the proportion of all offenders who install interlocks will increase. Currently, the extent to which courts managing interlock programs are also using BAC-monitoring systems like SCRAM™ and IN-HOM™ to monitor DUI offenders is unknown, but there is a trend for interlock providers to diversify into other electronic monitoring systems. It is probable that most providers will soon be able to deliver “full-service systems” for the courts in which they provide both the interlock and alcohol monitoring and, if it becomes available, a system for monitoring driving at comparable costs to the offender. The court then would have the discretion to use the most appropriate sanction for each case and move offenders from one program to another based on their performance (Voas et al. 2011). Ultimately, the offender might be given a choice of one of the three sanctions.

## Use of Electronic Health Technologies to Increase the Effectiveness of the Interlocks While on the Vehicle

In concept, the interlock prevents recidivism by making it impossible for offenders with a positive BAC to start their vehicles. Experience has demonstrated that the device does not achieve a 100 percent reduction because offenders find ways to circumvent the units installed on their vehicles or they use non–interlock-equipped cars. In a sample of New Mexico drivers using interlocks, Roth and colleagues (2007) found that 1.9 percent of offenders were rearrested for DUI while driving vehicles without an interlock. That was 76 percent of all the interlocked offenders rearrested. This is a major unsolved problem for interlock programs. Efforts to detect the use of a non–interlock-equipped vehicle primarily have been limited to requiring the interlock provider to check odometer readings at service visits and flag low driving mileage in their reports. It is unclear what actions are being taken based on such reports. The addition of face and photo identification to the interlock system does provide a means of detecting and deterring the swapping of cars with a spouse or family member who does not drink. When driving of a non-interlocked car is suspected, such identification systems also provide a method for monitoring drinking. Courts can require the offender to provide interlock breath tests on days when the vehicle is not driven to ensure that if driving another vehicle, he or she will not be impaired.

A lessor problem is the attempt to circumvent the unit by tampering with the electric power supply or by introducing bogus breath samples from passengers or using balloons or other storage devices. Most such efforts are detected or defeated by the anti-circumvention systems (recently enhanced by photo identification systems) in the interlock unit. Requirements for regular service visits, where attempts to circumvent will be detected, seem to be effective in discouraging tampering with the unit. The ability of providers to monitor the integrity of the devices recently has been enhanced by the provision of real-time Wi-Fi notification of violations to the program manager. Thus, the addition of new technologies such as Wi-Fi and photo identification systems have promise for further reducing offenders’ recidivism while the interlock is installed.

## Use of Electronic Health Technologies to Extend Time on the Interlock

The data-logger system, which records all BAC tests and engine ignitions, originally was added as a component of the interlock to detect circumvention attempts through the instances when the motor started without a breath test. Research (Marques et al. 2001, 2003*a*,*b*) has demonstrated that the number of lockouts experienced during the period the interlock is on the vehicle is predictive of recidivism after removal of the device. This has revealed an additional important health benefit of the interlock: the information it provides on the offender’s ability to adapt to the ban on driving when drinking. Lockouts appear to provide a screening device for identifying drivers at high risk for future impaired driving. Based on the relationship of lockouts to recidivism, some State legislatures have enacted laws providing for the extension of time on the interlock for offenders with high numbers of lockouts. Generally, these laws require an extension based on a fixed number of lockouts or specify that the last few months of the interlock period must be lockout free (Fiedler et al. 2012; Mayer 2014). This data-logger technology, which provides a method for monitoring driving behavior, establishes a basis for implementing automated adjustments to the length of the interlock experience based on offender performance. However, the practical effectiveness of extending time on the interlock has yet to be demonstrated.

**Figure 1:**
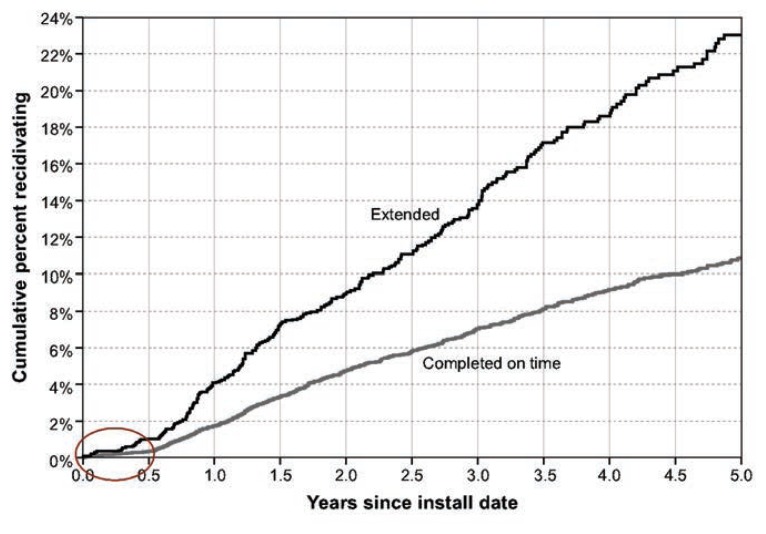
Cumulative recidivism over the 5-year period following the installation of the interlock for first DUI offenders in the Florida interlock program. Circled area indicates the 6-month period on the interlock following which they were fully relicensed.

Evaluating such programs will be difficult, as those who receive the added sanction are selected because of an excessive number of lockouts, which is associated with a high risk of recidivating. Thus, the offenders receiving extension will be a biased group with a higher-than-normal risk of recidivating. This confound is illustrated in a recent study by Voas and colleagues (2013) of first-time DUI offenders in the State of Florida, which requires time on the interlock as a prerequisite to reinstatement of regular driving privileges. Figure 1 shows the 5-year cumulative recidivism following interlock installation for first offenders who completed their prescribed 6 months on the interlock normally compared with those who were extended up to 12 months based on having three or more interlock violations. Six months (circled in the lower left-hand corner) is the required time on the interlock. Following that period, when there was little or no recidivism, those who completed normally, on time, and were reinstated, had a 2 percent per year recidivism rate, whereas those who were extended had a 4 percent rate. This finding suggests that basing the extension on the number of lockouts results in the identification of high-risk offenders. The benefit of extending the high-risk group is unclear; however, because extending the lockout confounds the offender’s level of risk with the potential benefit of extended time on the interlock. The difference between those who completed the lockout period on time and those who were extended does point to an opportunity, described below, to intervene with this high-risk group to reduce their post-interlock recidivism.

## Use of Technology to Extend the Effectiveness of Interlocks Beyond Removal

As described above, the time spent on the interlock provides a useful test period for measuring the participants’ ability to adapt their drinking and their driving behaviors to the limits imposed on them. The interlock data system captures this experience (Marques et al. 2001, 2003*a*,*b*). The transition to the restriction imposed by the interlock must be met in one of three ways: (1) reducing alcohol consumption, (2) reducing driving, or (3) managing the scheduling of each to avoid the conjunction of the two. It is reasonable to hypothesize that offenders coping with the increased restrictions imposed by the interlock are motivated to preserve their habitual consumption and driving levels, drinking environments, and drinking associates as much as possible. Presumably, they will attempt to defend those behaviors by managing their combination of drinking with driving to avoid an ignition lockout rather than reducing either driving or alcohol consumption. To date, there have been no in-depth studies of the changes in the drinking or the driving practices by individual offenders coping with the interlock’s restrictions.

## Adaptation to the Interlock: Evidence of Learning

Although limited information exists on how offenders adjust their drinking and driving to accommodate to the interlock, evidence clearly shows that the frequency of lockouts is reduced over the time the unit is on the offender’s vehicle (Vanlaar et al. 2010; Voas et al. 1999). This suggests that offenders learn improved safety behavior by the time they become eligible to remove the interlock. Of special interest in this learning process is that the safety gains seem to be achieved without a major change in the amount of driving, as indicated by the number of vehicle starts recorded by the interlock (Fiedler et al. 2012; Marques et al. 2001). Moreover, there is initial evidence that those gains are achieved without significant reductions in drinking. Marques and colleagues (2010) collected the blood, hair, and urine of DUI offenders participating in an interlock program in order to measure 6 different alcohol biomarkers such as ethylglururonide (EtG) and phosphatidylethanol (PEtH). These markers can provide a record of drinking ranging from days to several months. Samples were taken at the time of enrollment and again upon removal of the interlock. Using these biomarker measures on 300 (both first and multiple) DUI offenders in Canada, they found that although the offenders controlled their drinking to avoid lockouts, their total alcohol consumption, as measured by alcohol biomarkers, did not change while they were on the interlock. This suggests that they were successful in rearranging drinking in relation to their driving to accommodate the interlock without reducing their total consumption. Thus, recent evidence indicates that offenders can accommodate to the interlock both in their driving and in their drinking without marked reductions in either activity. However, this behavior does not continue into the post-interlock period, as recidivism rises markedly once the device is removed, as shown in figure 1 from Voas and colleagues (2013).

## Does Integrating Treatment With Interlocks Extend Their Effectiveness?

This finding that offenders adapt to the interlock without reducing their drinking suggests that an opportunity exists to extend the benefits of the interlock beyond de-installation. The studies of the extent to which DUI offenders exhibit signs of alcohol use disorders, together with the indication that they do not reduce total consumption while on the interlock, strongly suggest the efficacy of treatment programs targeting the offenders’ basic drinking problems. Treatment providers could use the electronic performance data collected by the interlock to tailor their programs to the needs the offenders. Aside from providing an overall view of the client’s ability to control his or her drinking, reviewing the data with the client also may be useful in breaking through denial and exploring healthful behavioral changes. For example, a pilot joint interlock and motivational intervention program was undertaken between 2000 and 2003 in Texas (Timken and Marques 2001*a*,*b*). Participants were presented with their monthly interlock records and were guided to finding actions that would avoid future failed BAC tests. That program has now been “manualized” and, as of 2011, is available to DUI offenders in interlock programs in Colorado (Timken et al. 2012*a*,*b*). Since 2008, Florida has combined counseling and treatment programs that are keyed to interlock performance. As the number of lockouts increase, so do the number of interventions (Voas et al. 2013). The possibility of implementing such integrated programs is limited in States that require treatment completion prior to interlock installation, or when the sharing of interlock data with treatment providers is limited (Voas et al. 2014).

**Figure 2:**
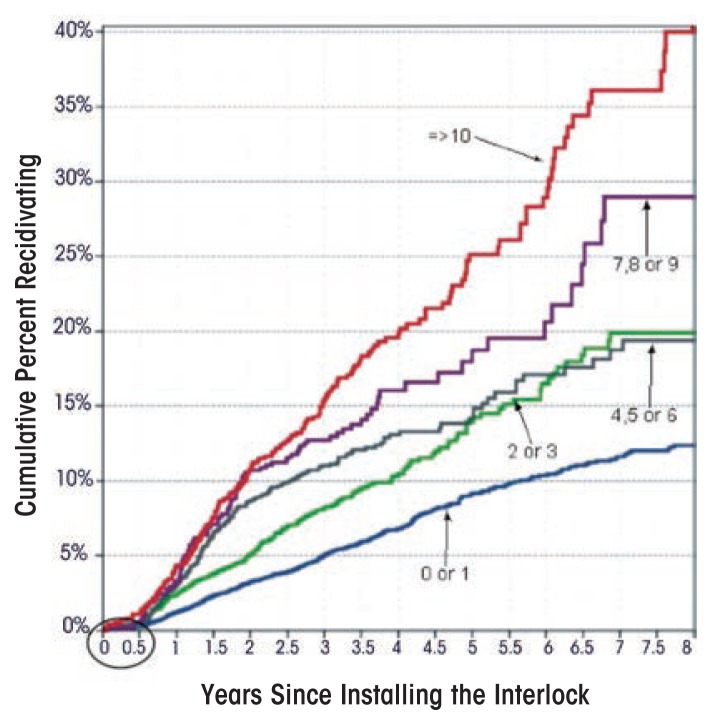
Relationship of the number of lockouts experienced by offenders while the unit is on their vehicle for 6 months (circled area) to their recidivism over the following 7 years.

The potential benefit for integrating education and treatment programs with the interlock is illustrated in figure 2 taken from the study by Voas and colleagues (2013). That study tracked the recidivism of 19,959 first-DUI offenders in the Florida interlock program over a 5-year period. The participants were stratified based on the number of lockouts they experienced in the 6 months (circled in the lower left-hand corner of the figure) that they had the device on their vehicles. Cumulative recidivism survival curves were calculated for each of the five lockout levels. The strong relationship between lockouts and recidivism following removal is clear. Also, it is apparent that this electronic monitoring system identifies those offenders who are most in need of a health intervention. Given the research evidence that offenders on interlocks are able to manage their drinking to avoid lockouts and the usefulness of lockouts in identifying the offenders most in need of treatment, the potential for integrating heath interventions while the offender is on the interlock deserves increased attention.

## The Role of Electronic Technology in the Emerging Criminal Justice System Model for DUI Offenders

Over the last two decades, the high cost and limited effectiveness of incarcerating impaired-driving offenders has led to the implementation of specialized DUI courts. Those courts are modeled on a unique program developed for managing drug offenders. DUI courts give offenders who are facing substantial jail sentences an opportunity to take part in a treatment and monitoring program that, if successfully completed, results in substantially reduced jail sentences. Emphasis is placed on replacing jail with monitoring programs, which are paid for by the offender or provided at low cost to the government. Minimized jail time is retained as a sanction for failure to conform to the monitoring requirements. The development of electronically monitored home confinement, alcohol consumption monitors (such as the SCRAM™ and IN-HOM™ devices), and vehicle alcohol interlocks allow the incapacitation of nonviolent substance abusers. Such community correction programs can be implemented at a much lower cost and for longer periods of time than incarceration. In such community programs, the offender is able to work and, thereby, finance much of the control program. Further, the correction programs are applied within the normal living environment where the targeted consumption adjustments occur and where both professional treatment assistance and family support are most available.

In this evolving system, sanction alternatives are maximized to increase flexibility in meeting offender needs. In the past, sanction alternatives were limited to jail, fines, and license suspension. New technologies provide programs based on BAC measurement and include electronically monitored home confinement and vehicle interlock programs. Courts now have many more options for managing impaired drivers. This is leading to a new and comprehensive model for managing impaired-driving offenders. That model emphasizes adaptive sentencing that more effectively takes into account the nature of the offender’s problem and treatment needs in addition to the seriousness of the offense. Courts now have more than a single option, such as jail time or license suspension, and can opt instead to use behavioral triage to create performance-based sanction programs where conformance to sanction requirements is rewarded by less intrusive monitoring and shorter sentences (see Voas et al. 2011 for a full description of this emerging model). A comprehensive interlock program fits that model by combining a relatively intrusive and confining set of sanctions such as home confinement or BAC monitoring with the less intrusive interlock providing a set of graded options. The more intrusive options can be used with the more serious offenders or as an option for those attempting to avoid or circumvent the interlock. Offenders can earn a reversion to the less intrusive interlock by demonstrating their ability to conform to the requirements of the monitoring systems and eventually, following a period on the interlock, have their licenses restored. When combined with an integrated treatment system this performance based system holds promise for extending the effectiveness of interlocks to the lifetime of driving that follows their removal.
